# Health problems and utilization of health services among Forcibly Displaced Myanmar Nationals in Bangladesh

**DOI:** 10.1186/s41256-021-00223-1

**Published:** 2021-10-11

**Authors:** Lal B. Rawal, Kie Kanda, Tuhin Biswas, Md. Imtiaz Tanim, Padam Kanta Dahal, Md. Rajibul Islam, Tarique Md. Nurul Huda, Tahmina Begum, Berhe W. Sahle, Andre M. N. Renzaho, Iqbal Anwar

**Affiliations:** 1 School of Health Medical and Applied Sciences, Collage of Science and Sustainability, Central Queensland University, Sydney Campus, Sydney, Australia; 2 Physical Activity Research Group, Appleton Institute, Central Queensland University, Wayville, Australia; 3 Translational Health Research Institute, Western Sydney University, Sydney, Australia; 4 School of Nursing and Midwifery, Western Sydney University, Sydney, Australia; 5 The University of Queensland, Brisbane, Australia; 6 mPower Social Enterprises Ltd., Dhaka, Bangladesh; 7 Health Intervention and Technology Assessment Program (HITAP), Nonthaburi, Thailand; 8 Infectious Diseases Division, icddr,b, Dhaka, Bangladesh; 9 Health Systems and Population Division, icddr,b, Dhaka, Bangladesh; 10 School of Nursing and Midwifery, Deakin University, Geelong, VIC Australia; 11 Centre for Quality and Patient Safety Research (QPS), Alfred Health Partnership, Melbourne, VIC Australia; 12 Melbourne School of Population and Global Health, University of Melbourne, Melbourne, VIC Australia; 13 School of Medicine, Western Sydney University, Sydney, Australia

**Keywords:** Rohingya refugees, Bangladesh, Family planning, HIV/AIDS, Health services

## Abstract

**Background:**

Access to and utilization of health services have remained major challenges for people living in low- and middle-income countries, especially for those living in impaired public health environment such as refugee camps and temporary settlements. This study presents health problems and utilization of health services among Forcibly Displaced Myanmar Nationals (FDMNs) living in the southern part of Bangladesh.

**Methods:**

A mixed-method (quantitative and qualitative) approach was used. Altogether 999 household surveys were conducted among the FDMNs living in makeshift/temporary settlements and host communities. We used a grounded theory approach involving in-depth interviews (IDIs), focus group discussions (FGDs), and key informant interviews (KIIs) including 24 IDIs, 10 FGDs, and 9 KIIs. The quantitative data were analysed with STATA.

**Results:**

The common health problems among the women were pregnancy and childbirth-related complications and violence against women. Among the children, fever, diarrhoea, common cold and malaria were frequently observed health problems. Poor general health, HIV/AIDS, insecurity, discrimination, and lack of employment opportunity were common problems for men. Further, 61.2% women received two or more antenatal care (ANC) visits during their last pregnancy, while 28.9% did not receive any ANC visit. The majority of the last births took place at home (85.2%) assisted by traditional birth attendants (78.9%), a third (29.3%) of whom suffered pregnancy- and childbirth-related complications. The clinics run by the non-governmental organizations (NGOs) (76.9%) and private health facilities (86.0%) were the most accessible places for seeking healthcare for the FDMNs living in the makeshift settlements. All participants heard about HIV/AIDS. 78.0% of them were unaware about the means of HIV transmission, and family planning methods were poorly used (45.2%).

**Conclusions:**

Overall, the health of FDMNs living in the southern part of Bangladesh is poor and they have inadequate access to and utilization of health services to address the health problems and associated factors. Existing essential health and nutrition support programs need to be culturally appropriate and adopt an integrated approach to encourage men’s participation to improve utilization of health and family planning services, address issues of gender inequity, gender-based violence, and improve women empowerment and overall health outcomes.

## Introduction

The increasing number of displaced populations has become one of the major human rights and health problems worldwide [1]. The number of those forcibly displaced internally or to the neighbouring countries has increased from 20 million in 2000 to 41.1 million in 2010 and 79.5 million in 2019, of whom at least 26 million are stateless [1, 2]. The major reasons for such forced situation include violent conflict and persecution, compounded by rising food insecurity, natural disasters, and poor governance. Forcibly displaced people generally face human rights and health-related issues such as lack of access to basic healthcare, education, and employment opportunities as well as limited freedom of movement. They live in precarious situations characterized by the denial or loss of nationality, exclusion and discrimination, and oppression [2–4].

The increasing deterioration of security in some countries of the Asia and Pacific regions has resulted in increased internal and cross border displacements. These regions currently host over eight million displaced people including 3.5 million refugees, 2.7 million internally displaced persons (IDPs) and 1.6 million stateless people who are predominantly refugees from Afghanistan and Myanmar [1]. The Rohingya people of Myanmar are among the largest groups of stateless people in the world [5] accounting for one in seven of the global stateless population [6]. The majority of Rohingya refugees are not yet considered citizens by the Myanmar Government [7, 8] and a large number of them have been fleeing Myanmar to nearby countries primarily Bangladesh, Malaysia and Thailand to avoid conflict and persecution. Bangladesh has been a preferred destination for Rohingya refugees due to its close proximity and matching culture and religion [9, 10]. Since 1948, Bangladesh has hosted a majority of Rohingya refugees and Forcibly Displaced Myanmar Nationals (FDMNs) who entered Bangladesh in three major influxes in 1978, 1992 and 2016–2017 [11, 12].

An estimated 32,000 Rohingya refugees have been recognized as registered refugees by the United Nations High Commission for Refugees (UNHCR) and reside in two official camps supported by the UNHCR in the Cox’s Bazar district of Bangladesh [13]. However, over 300,000 Rohingya refugees continue to reside in unofficial makeshift camps [13]. Recent increases in violence in Myanmar have drawn international attention and have caused large numbers of Rohingya refugees to cross the border into Bangladesh, making the total number of new arrivals to 693,000 as of June 2018 [14, 15]. With more than two decades of camps and settlements, and the current increased number of Rohingya refugees in Bangladesh, it has become more serious problem to the Bangladesh Government and the international community [9, 14].

Despite the improvement in the overall health of people in low- and middle-income countries (LMICs), access to proper health and nutrition remains a major challenge in many LMICs including Bangladesh. Globally, there are over 400 million people who do not have access to essential health care services and majority of them live in LMICs [16, 17]. For those living in special circumstances, such as migrants, displaced persons, and refugees, the accessibility to essential health and nutrition services remains even more critical [18]. Whether living in the refugee camps, the makeshift settlements, or the local communities, FDMNs have been in difficult living conditions marked by inadequate access to basic needs, exposure to violence, poor access to health services, restricted movement, local hostility, and various forms of discrimination [9]. The children in the refugee camps are vulnerable to malnutrition and infectious diseases [19].

Rohingya refugees living in the refugee camps supported by UNHCR are provided with humanitarian aid including regular supply of food and water, health services, education and shelter [14]. However, the other FDMNs residing in makeshift camps have no access to such humanitarian assistances, but receive basic services coordinated through the International Organization for Migration (IOM) in accordance with the Bangladesh National Strategy on Myanmar Refugees and Forcibly Displaced Myanmar Nationals. There are also some FDMNs who live within the local communities nearby, but no official figure is recorded yet. Under the framework of the Bangladesh National Strategy, IOM has been mandated to undertake and coordinate humanitarian efforts for the FDMNs particularly on health, water and sanitation, and gender-based violence. Since September 2013, IOM in partnership with other organizations has been implementing the humanitarian interventions to increase access to health services to both FDMNs and local communities. Evidence describing the health problems, access to and utilization of health services among FDMNs and local communities remains scarce. Therefore, this study aimed to characterize health problems, access to and utilization of health services among FDMN living in Teknaf and Ukhia Upazilas (sub-districts) of the Cox’s Bazar district, Bangladesh.

## Methods

### Study design and population

A mixed methods (qualitative and quantitative methods) approach was used for data collection, data processing and analyses of this study. The FDMNs residing in the makeshift settlements located in Taknaf and Ukhia Upazilas (sub-districts) and the local host communities, as well as the local people in the host communities of two sub-districts, were considered as participants in this study.

### Sampling strategy and sample size

**Fig. 1:**
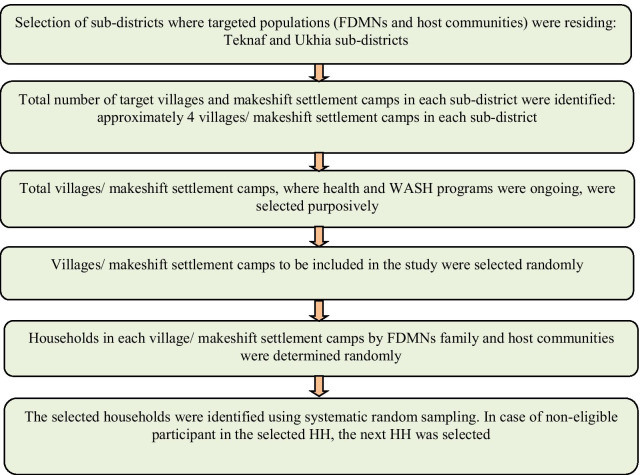
Flow chart describing sampling strategy and data collection process used in this study

A multi-stage stratified random sampling was used to determine the study areas including sub-districts, makeshift settlement camps, and households in both two sub-districts (Fig. 1). In the first stage, two sub-districts where the FDMNs were residing were selected purposively. In the second stage, the total number of target villages and makeshift settlements in each sub-district where FDMNs were residing was listed and four makeshift settlements in each sub-district were selected randomly. In the third stage, the total number of households in each makeshift settlement was identified by using the record provided by IOM. The total sample of households required for this study was determined based on the estimated proportion of pregnant women accessing comprehensive essential obstetric care (17% of live births) [20]. In the final stage, the households were identified by using a systematic random sampling strategy. The minimum sample size for quantitative study was estimated at 978 women having at least one child below five-year-old. Eventually, a total of 999 women participated in the quantitative study. The participants for the qualitative interviews and focus group discussions were identified from the same villages where quantitative data were collected. Women, men, camp management committee chairmen, NGO representatives, health workers, community volunteers, and community/ward chairpersons were included in the qualitative study.

### Data collection

In terms of quantitative data collection, 16 trained field research assistants, who understood the local language spoken in the study areas were involved. A three-day training was provided for the research assistants prior to the field data collection, and the data collection tools were tested in the field. Along with the technical contents of the tools, the cultural sensitivity and ethical conduct of the study while collecting data, were emphasized. During the data collection, two field coordinators oversaw the surveys and ensured the quality of data. Altogether 999 surveys were conducted. The mothers with a child below five years were the main participants. No refusal to the survey questionnaire was reported.

In terms of qualitative data collection, altogether 24 in-depth interviews (IDIs), 10 focus group discussions (FGDs) and 9 key informant interviews (KIIs) were performed by a separate group of trained research assistants, who had an educational background in anthropology and had prior experience of conducting qualitative interviews. At least two IDIs and two FGDs in each area were conducted among the men and women participants. The KIIs included chairmen of the camp management committees, members of the village development committees, health volunteers, doctors, and local community ward members. During IDIs, KIIs and FGDs, notes were taken, and voices were digitally recorded after obtaining the informed verbal consent. Research assistants provided translations and summaries (in English) to the supervisors and study team within a few days following the data collection. The field data collection took place during the months of August and September 2016.

### Data analysis

The quantitative data were entered into the SPSS Statistical software version 16 and data were analysed with STATA. Descriptive analysis was performed to describe the current health situation, followed by cross tabulation and Chi-square test to determine the association between general characteristics and health outcomes. The study variables included (1) independent: ethnicity, age, education, occupation of women and household head and household income (2) dependent variables: general health problems, health problems of women and children, ANC, delivery and PNC, family planning methods knowledge and use, HIV and TB knowledge, gender-based violence, and health services access and use. *P* value less than 0.05 was considered as statistically significant.

Given the nature of the qualitative data, we used the framework method of analysis following Gale et al*.* [21]. The framework method is appropriate for comparing and contrasting large scale textual data across cases (in this case, the IDIs, FGDs and KIIs information) and synthesizing the data in a thematic way (‘a holistic, descriptive overview’). This approach identifies commonalities and differences in qualitative data before focusing on relationships between different parts of the data, thereby seeking to draw descriptive and/or explanatory conclusions clustered around themes, and now is used widely in health research [21–23].

All interviews and notes taken in Bangla were translated into English. A sample of transcripts was read and re-read by the study team members independently to develop an initial coding matrix of themes and categories. This was discussed, refined, and agreed upon before the remainder of the transcripts were prepared and analyzed using the agreed coding framework. All translated transcripts were compiled and read multiple times to facilitate data familiarization. Then the data were coded, an analysis framework was developed in a tabular form with themes and sub-themes, and the analytic matrix was populated with compiled data. Finally, data validation and interpretation were done.

## Results

### General characteristics of the participants and accessible health facilities

The majority of the study participants were FDMNs (72.7%) either living at makeshift settlements or in the host community and a quarter (26.8%) were Bengali populations (Table 1). They were Muslims, predominantly married and living with a husband, unemployed (mainly housewives), with a mean age (standard deviation-SD) of 25 years (± 6 years). The majority of the FDMNs in makeshift settlements (76.9%) as well as the FDMNs living in the host communities (57.1%) said that the NGO clinics were the most accessible places for seeking health care services (Table 1). However, the government health facilities were most accessible places for the local communities. Private pharmacies were the other most accessible places for those living in makeshift settlements. The practice patterns of using health facilities when they fall sick was aligned to the ones they expressed as the most accessible health facilities.

**Table 1:** General characteristics of the participants and accessible health facilities (n = 999)

Socio-demographic information	FDMN in makeshift settlement n (%)	FDMN in host community n (%)	Host community n (%)	Total n (%)
*Ethnicity*				
Bengali	14 (3.3)	41 (11.5)	212 (99.1)	267 (26.7)
FDMNs	411 (96)	316 (88.5)	0 (0)	727 (72.8)
Rakhaine/Murma/Chakma/Others	3 (0.7)	0 (0)	2 (0.9)	5 (0.5)
*Religion*				
Islam	426 (99.5)	356 (99.7)	213 (99.5)	995 (99.6)
Others	2 (0.5)	1 (0.3)	1 (0.5)	4 (0.4)
*Age*				
≤ 19 years	64 (15)	50 (14.1)	12 (5.7)	126 (12.7)
20–29	273 (63.8)	217 (61.3)	145 (69.4)	635 (64.1)
30–39	82 (19.2)	79 (22.3)	47 (22.5)	208 (21)
40 and above	9 (2.1)	8 (2.3)	5 (2.4)	22 (2.2)
*Marital status*				
Married (living with husband/family)	400 (93.5)	337 (94.4)	209 (97.7)	946 (94.7)
Separated/widow (living alone)	28 (6.5)	20 (5.6)	5 (2.3)	53 (5.3)
*Occupation of the women*				
Housewife/unemployed	343 (96.1)	207 (96.7)	948 (94.9)	343 (96.1)
Employed (small business, daily wager)	14 (3.9)	7 (3.3)	51 (5.1)	14 (3.9)
*Occupation of the household head*				
Unskilled labourer/daily wages	345 (80.6)	158 (44.3)	106 (49.5)	609 (61)
Skilled labourer/service	19 (4.4)	25 (7)	52 (24.3)	96 (9.6)
Small business	38 (8.9)	12 (3.4)	35 (16.4)	85 (8.5)
Farming/Fishing/agricultural worker	14 (3.3)	157 (44)	20 (9.3)	191 (19.1)
Unemployed	12 (2.8)	5 (1.4)	1 (0.5)	18 (1.8)
≤ 4000 (US$ 51)	94 (22.8)	76 (21.6)	19 (9.1)	189 (19.4)
4000–5000 (US$ 51–64)	64 (15.5)	74 (21)	14 (6.7)	152 (15.6)
5001–6000 (US$ 64–77)	142 (34.4)	112 (31.8)	48 (23)	302 (31)
6001–9000 (US$ 77–115)	56 (13.6)	53 (15.1)	25 (12)	134 (13.8)
≥ 9000 (US$ 115 and above)	57 (13.8)	37 (10.5)	103 (49.3)	197 (20.2)
*Accessible health facilities*				
Government health facility*	77 (18.0)	143 (40.1)	59 (27.6)	279 (27.9)
NGO Clinics	329 (76.9)	204 (57.1)	48 (22.4)	581 (58.2)
Private clinics	159 (37.1)	204 (57.1)	184 (86.0)	547 (54.8)
*Health facilities frequently visited*				
Government health facility*	62 (14.5)	127 (35.6)	57 (26.6)	246 (24.6)
NGO Clinic	310 (72.4)	190 (53.2)	39 (18.2)	539 (54.0)
Private health facility	148 (34.6)	211 (59.1)	182 (85.0)	541 (54.2)

^*^Community clinic; Satelite clinic/ expanded program of immunization outreach clinic; Upazila health and family welfare centre; Upazila health complex; Maternal and child welfare centre; and District hospital

### Access to and use of health services

In Leda Makeshift Settlement (LMS), the closest formal health facility available was the government community clinic, which was about 20 min walking distance from the makeshift settlement. However, most of the female and male participants of LMS stated that they preferred to visiting private pharmacies located within the settlements. The participants from LMS mentioned that there used to be a health facility run by an NGO within LMS, however it was closed in 2013. Since then, there has been a big challenge in accessing health services properly. One of the female participants from LMS said the following:

It takes about 20 min by bus to get to the government hospital. We also face long que to receive the care after reaching there. Therefore, we prefer getting medicine from the private pharmacy located within the camp. – IDI-F- L makeshift camp.

#### ANC, pregnancy care and PNC

Over two in five (40.5%) women knew that the minimum number of ANC required per episode of pregnancy is four or over; however, almost one in five (18.6%) admitted that they did not know the number of ANC visits required during the pregnancy. Regarding the danger signs of pregnancy which they suffered during their last pregnancy, almost one-third (29.3%) mentioned that they had at least one of them during their last pregnancy (Table 2). NGOs’ clinics were the most frequently visited health facilities for seeking care for danger signs during the pregnancy. The majority of the female participants received ANC at least twice or more (61.2%) in their last pregnancy, but again almost a third (28.9%) did not receive any ANC visit. The majority of the last births took place at home (85.2%) and were assisted by traditional birth attendants (78.9%). Regarding the postnatal care and practice, over a third (41.6%) received PNC after their last childbirths (Table 2). In terms of additional nutritious foods required during the pregnancy, both the men and women from the two makeshift settlements said that they were quite aware of the needs during pregnancy; however, due to lack of money, it was quite hard for them to afford those food. One male participant from a makeshift settlement stated that:

I know during pregnancy, wife needs good food like meat, fish, vegetable and fruits, but I am a poor man; I hardly manage our living, so I can’t afford these good foods for my wife. She eats like everyone else in the family. – IDI-M- K makeshift camp.

**Table 2:** Knowledge and practices on ANC, pregnancy, delivery and PNC

ANC, pregnancy and PNC care	FDMN in makeshift settlement n (%)	FDMN in host community n (%)	Host community n (%)	Total n (%)	*P* value
*Knowledge on required ANC visits in one pregnancy*					
2 ANC visits	8 (1.9)	9 (2.5)	3 (1.4)	20 (2)	< 0.001
3 ANC visits	119 (27.8)	153 (42.9)	116 (54.2)	388 (38.8)	
4 and more ANC visits	246 (57.5)	92 (25.8)	67 (31.3)	405 (40.5)	
Don’t know	55 (12.9)	103 (28.9)	28 (13.1)	186 (18.6)	
*Episodes of ANC visits received in last pregnancy*					
Did not do ANC visit	51 (11.9)	187 (52.4)	51 (23.8)	289 (28.9)	< 0.001
One ANC visit	25 (5.8)	47 (13.2)	27 (12.6)	99 (9.9)	
2 ANC visits	34 (7.9)	37 (10.4)	36 (16.8)	107 (10.7)	
3 ANC visits	148 (34.6)	69 (19.3)	75 (35.0)	292 (29.2)	
4 or more ANC visits	170 (39.7)	17 (4.8)	25 (11.7)	212 (21.2)	
*Common places where ANC service was received*					
Government health facility	41 (10.9)	74 (44)	54 (33.1)	169 (23.9)	< 0.001
NGO run clinic	329 (87.5)	99 (58.9)	88 (54)	516 (73)	
Private health facility	27 (7.2)	12 (7.1)	36 (22.1)	75 (10.6)	
Person seen for last ANC visit					
Trained health personnel^a^	349 (92.6)	162 (95.3)	149 (91.4)	660 (93)	0.003
Non-trained personnel^b^	70 (18.6)	11 (6.5)	27 (16.6)	108 (15.2)	
*Number of TT vaccine during last pregnancy*					
TT vaccine given	336 (78.5)	291 (81.5)	193 (90.2)	820 (82.1)	0.001
Mean numbers	2	3	2	2	
*Knowledge about danger signs of pregnancy*^c^					
Not known at all	48 (11.2)	60 (16.8)	20 (9.3)	128 (12.8)	0.028
At least two danger signs	203 (47.4)	176 (49.3)	105 (49.1)	484 (48.4)	
Known at least three or more danger signs	177 (41.4)	121 (33.9)	89 (41.6)	387 (38.7)	
*Suffered any complications during last pregnancy/birth*					
Yes, suffered complications^d^	124 (29.0)	87 (24.4)	81 (37.9)	292 (29.2)	0.003
No, did not suffer complications	304 (71.0)	270 (75.6)	133 (62.1)	707 (70.8)	
*Health facility visited for complications management*					
Government health facility	23 (19)	17 (20.7)	19 (24.1)	59 (20.9)	< 0.001
NGO run clinic	84 (69.4)	31 (37.8)	18 (22.8)	133 (47.2)	
Private health facility	14 (11.6)	34 (41.5)	42 (53.2)	90 (31.9)	
Place of last birth					
Home	326 (76.2)	344 (96.4)	181 (84.6)	851 (85.2)	< 0.001
Government health facility	15 (3.5)	6 (1.7)	19 (8.9)	40 (4)	
NGO Clinic	83 (19.4)	3 (0.8)	8 (3.7)	94 (9.4)	
Private health facility	4 (0.9)	4 (1.1)	6 (2.8)	14 (1.4)	
*Person assisted for delivery at health facility*					
Trained health personnel	169 (75.4)	27 (30)	39 (38.6)	235 (56.6)	< 0.001
Non-trained personnel	85 (37.9)	64 (71.1)	63 (62.4)	212 (51.1)	
Trained health personnel	169 (75.4)	27 (30)	39 (38.6)	235 (56.6)	
*Number of last PNC visits*					
1 PNC visit	60 (26.8)	32 (35.6)	36 (35.6)	128 (30.8)	0.362
2 PNC visits	79 (35.3)	26 (28.9)	28 (27.7)	133 (32)	
3 PNC visits	53 (23.7)	22 (24.4)	19 (18.8)	94 (22.7)	
4 or more PNC visits	32 (14.3)	10 (11.1)	18 (17.8)	60 (14.5)	
*Places for PNC check-up*					
Government health facility	15 (6.9)	9 (10.7)	18 (18.6)	42 (10.6)	< 0.001
NGO run clinic	169 (77.9)	21 (25)	13 (13.4)	203 (51)	
Private health facility	33 (15.2)	54 (64.3)	66 (68)	153 (38.4)	

^a^Medical doctor, Nurse/midwife/paramedic, Family welfare visitor, Community skilled birth attendant, Medical Assistant/SACMO, Health Assistant, Family welfare assistant

^b^Traditional birth attendant, unqualified doctor, NGO worker

^c^Danger signs of pregnancy (per vaginal bleeding, severe headache, leaking of fluid from the vagina, Abdominal pain, sudden blur vision, high fever, convulsion, prolonged labor)

^d^Complications during the last pregnancy/birth: convulsion, difficulty in breathing/fast breathing, infection of umbilical stump, unable to suck or difficulty in feeding, low/high body temperature, lathery

Despite almost two-third receiving ANC visits, most of them gave birth at home assisted by traditional birth attendants. The participants of the FGDs and IDIs provided a number of reasons behind this, including traditional practices to giving birth at home, religious values, unavailability of trained midwives at the nearest health centre, hesitation to visit health facility, and lack of financial resources to afford health care cost. Several participants also stated that a health facility which was run by an NGO within the makeshift camp was closed, therefore they did not have easy access to get assistance for giving birth. One of the participants explained that:

We used to go to the hospital which was inside the settlement. However, it was closed two years ago. Since then, we feel somehow difficult to go to the nearest health centre which is outside the settlement. Some people still go to the health centre though- FGD-F- L makeshift camp.

#### Neonatal and child health

The result showed that mothers were aware of at least two danger signs of the new-born baby including difficulty in breathing/fast breathing and low/high body temperature. Regarding the vaccination status of the children, the vaccination card was checked. Over a half (52.2%) of the children had completed the all the routine vaccinations. Almost all women were familiar with the primary symptoms and treatment of diarrhoea (Table 3). The qualitative results suggest that the common childhood diseases that occurred in the makeshift settlements included viral fever, common cold, pneumonia, diarrhoea, jaundice and malaria. It was reflected by a father of three children that:

It is not unusual that the children always suffer from diarrhoea. They can’t go to school, so they play outside in dirt all the time and eat things they should not. Also, we live in a place which is so much crowded, and enough water is not available. Thus, our children often get sick – IDI-M- L makeshift camp.

**Table 3:** Knowledge and practices on neonatal and child health care

Variables	FDMN in makeshift settlement n (%)	FDMN in host community n (%)	Host community n (%)	Total n (%)	*P* value
*Danger signs of new-born baby*					
Not known at all	29 (6.8)	34 (9.5)	14 (6.5)	77 (7.7)	0.289
Knows at least one sign	90 (21)	86 (24.1)	55 (25.7)	231 (23.1)	
Knows at least two or more signs	309 (72.2)	237 (66.4)	145 (67.8)	691 (69.2)	
Fed within one hour	145 (33.9)	92 (25.8)	72 (33.6)	309 (30.9)	0.034
Fed between 1 and 3 h	175 (40.9)	156 (43.7)	97 (45.3)	428 (42.8)	
Fed between 4 and 24 h	38 (8.9)	27 (7.6)	15 (7)	80 (8)	
Fed after 24 h	70 (16.4)	82 (23)	30 (14)	182 (18.2)	
*Given something to new-born for drink before initiating breast feeding*					
Yes, given	146 (34.1)	197 (55.2)	44 (20.6)	387 (38.7)	< 0.001
*Given anything to drink to new born within 3 days*					
Yes, given	192 (44.9)	219 (61.3)	62 (29)	473 (47.3)	< 0.001
*Age (in months) first provided additional food*					
Mean ± SD	4 ± 6	3 ± 7	5 ± 5	4 ± 6	< 0.001
Before 6 months	228 (54.9)	249 (70.9)	89 (43.2)	566 (58.2)	
After 6 months	187 (45.1)	102 (29.1)	117 (56.8)	406 (41.8)	
*Vaccination status*					
Vaccination completed	353 (82.5)	271 (75.9)	200 (93.5)	824 (82.5)	< 0.001
Not completed (still taking)	75 (17.5)	86 (24.1)	14 (6.5)	175 (17.5)	
*Having card for vaccination*					
Yes, card is available	181 (51.3)	122 (45)	127 (63.5)	430 (52.2)	< 0.001
*Knowledge on symptoms of diarrhea*^a^					
Not known at all	16 (18.0)	18 (5)	6 (2.8)	40 (4)	0.025
Knows at least 1 symptom	183 (18.0)	180 (50.4)	85 (39.7)	448 (44.8)	
Knows at least two or more symptoms	229 (15.9)	159 (44.5)	123 (57.5)	511 (51.2)	
*Those who could say about transmission of diarrhoea*					
Did not know	38 (45.0)	45 (12.6)	17 (7.9)	100 (10)	< 0.001
At least 1 way	120 (13.5)	135 (37.8)	56 (26.2)	311 (31.1)	
At least two or more ways	270 (17.7)	177 (49.6)	141 (65.9)	58 (8.9)	

^a^Symptoms of diarrhoea: frequent loose/watery stools, abdominal cramps/pain, presence of blood in the stool, dehydration, weakness/lethargy

#### Family planning, HIV and tuberculosis

Around 95% of the participants knew at least one modern family planning (FP) method. Over half of the women (54.9%) mentioned that they did not have any idea about the male FP methods at all. In terms of the use of FP methods, over a half (54.8%) of the participants were not using any FP methods. There were several reasons for not using FP methods: infrequent sexual intercourse (23.9%); wanting as many children as possible (15.3%); husbands or partners opposing to FP methods (14.6%); and religious brief (15.0%).

The majority of the respondents did not know about the means of transmission of HIV/AIDS, where only one in five (22.1%) mentioned sexual intercourse, and 5% agreed that contaminated needle prick might cause HIV/AIDS transmission (Table 4). More than a half of the female participants (53.6%) responded that they were not aware of the symptoms of tuberculosis (TB), whereas 41.9% answered coughing up blood and 13.5% coughing over three weeks. The results indicated that the female participants in the host community had a better level of knowledge on TB symptoms compared to the other two groups (Table 4).

**Table 4:** Knowledge and practices on family planning, HIV and tuberculosis

Knowledge and practices	FDMN in makeshift settlement n (%)	FDMN in host community n (%)	Host community n (%)	Total n (%)	*P* value
*Knowledge about FP methods for women*^a^					
Knew at least one modern FP method	406 (33.8)	338 (94.7)	200 (93.5)	944 (94.5)	0.750
Did now know	22 (19.0)	19 (5.3)	14 (6.5)	55 (5.5)	
*Knowledge about FP methods for men*					
Knows at least 1 method	219 (11.8)	118 (33.1)	106 (49.5)	443 (44.3)	< 0.001
Not known at all	209 (23.9)	239 (66.9)	108 (50.5)	556 (55.7)	
*Currently doing something or using any method/s to delay or avoid getting pregnant*					
Yes	189 (15.1)	151 (42.3)	111 (51.9)	451 (45.1)	0.073
No	239 (20.6)	206 (57.7)	103 (48.1)	548 (54.9)	
*Heard about the emergency contraception*					
Yes	84 (52.0)	52 (14.6)	31 (14.5)	167 (16.7)	0.103
No	344 (30.5)	305 (85.4)	183 (85.5)	832 (83.3)	
*Knowledge about transmission of HIV*					
Knows at least 1 approach how HIV transmitted	103 (48.0)	48 (13.4)	68 (31.8)	219 (22.0)	< 0.001
Not known at all	322 (30.9)	309 (86.6)	146 (68.2)	777 (78.0)	
*Knows the symptoms of tuberculosis*					
Knows at least 1 symptom	200 (13.5)	135 (37.9)	127 (59.3)	462 (46.4)	< 0.001
Not known at all	226 (22.1)	221 (62.1)	87 (40.7)	534 (53.6)	

^a^FP methods: permanent method (ligation), pills, injectable, IUDs/Cupper-T, implants, safe period, abstinence

Qualitative data show that the male participants in the makeshift settlements were not interested in using any forms of FP methods, and thus, women used FP methods, mainly contraceptive injections, or oral pills. Several male participants stated during FGDs that discomfort, sexual dissatisfaction, and religious belief against using the FP methods were the major reasons for not using FP methods. These ideas were reflected in the opinion of a male participant:

It is not right to use contraceptive method, because it is a sinful act. It means killing a baby. But people use contraceptive methods to keep some gap between two children, so that wife can rear children properly. But men here do not like using family planning methods. – FGD-M- L makeshift camp.

However, a few male participants were aware that sexual intercourse without protection like condom can increase the risk of unwanted pregnancies, sexually transmitted infections (STI) and HIV infection.

#### Gender-based violence

According to the quantitative and qualitative data, gender-based violence (GBV) was observed in the makeshift settlements. Almost all the female participants mentioned that they suffered from some forms of violence from their intimate partners. However, GBV by non-intimate partners is also a significant problem. FGDs revealed that violence mostly against female FDMNs happened in the makeshift settlements. The major forms of physical violence included physical assault with bare hand/slap, rape/forced sex, sexual harassment, and hitting with the solid object. Female FDMNs reported verbal violence including verbal insult and abuse. One of the female participants reported that:

My husband beat me even when I was pregnant; he even kicked me on my belly. I thought my unborn baby might have died. He beat me because I quarrelled with female neighbour. Fortunately, my mother-in-law came in and saved my life. – IDI-F- L makeshift camp.

In terms of seeking help for GBV, the women answered that family members and neighbour were the first to go to (33.6%), followed by seeking support and care from nowhere (25.8%), and seeking help from the police (23.6%) (Table 5). The qualitative data also validated these findings that in most cases the victims of GBV seek support from the family members, relatives, or neighbours. Some participants also informed the camp block management committee about the case. The male FDMNs also faced different forms of violence and discrimination in the makeshift settlements and in the host communities.

**Table 5:** Gender-based violence (multiple answers were obtained)

Gender based violence	FDMN in makeshift settlement N (%)	FDMN in host community N (%)	Host community N (%)	Total N (%)
*Major form of gender-based violence they suffered*				
Physical assault with bare hand	328 (76.6)	248 (69.5)	173 (80.8)	749 (74.9)
Rape/forced sex	218 (50.8)	179 (50.1)	88 (41.1)	485 (48.5)
Sexual harassment	106 (24.6)	54 (15.1)	48 (22.4)	208 (20.7)
Hitting with a solid object	61 (14.3)	50 (14.0)	39 (18.2)	150 (15.0)
Insulting comments/scolding	93 (21.5)	59 (16.5)	56 (26.2)	208 (20.7)
Threats of violence	61 (14.3)	50 (14.0)	30 (14.0)	141 (14.1)
Emotional/psychological abuse	72 (16.9)	70 (19.6)	47 (22.0)	189 (18.9)
*Common place of seeking help*				
Parents’/relatives house	188 (44.7)	83 (23.4)	61 (28.6)	332 (33.6)
Seeking care nowhere	70 (16.4)	133 (37.6)	53 (24.9)	256 (25.8)
Neighbours	74 (17.6)	44 (12.4)	24 (11.3)	142 (14.4)
Police	89 (21.1)	55 (15.5)	89 (41.8)	233 (23.6)
Hospital	4 (1.0)	6 (1.7)	1 (0.5)	11 (1.1)
Do not know where to go	29 (6.9)	30 (8.5)	10 (4.7)	69 (7.0)

## Discussion

In this study, we determined the overall health situation of the FDMNs living in the makeshift settlements in the Cox’s Bazar district of Bangladesh. We have provided the evidence base concerning the general health problems, access to and use of health services, as well as knowledge and practice on family planning, HIV/AIDs, TB and gender-based violence.

The findings of our study show that the common health problems among the FDMNs were varied by gender and age groups. The female FDMNs had a range of maternal and child health issues such as inadequate ANC and PNC, and childbirth at home assisted by untrained birth attendants. The male FDMNs had issues mainly in relation to discrimination, lack of employment opportunity, physical/verbal abuse, and insecurity. These findings are similar to the findings of previous studies which reported the problems and issues among the refugees and illegal migrants [1, 15, 24–27]. While women of refugee and asylum seeker background face several challenges to health care, a systematic review by Helen et al. (2020) reported that the community based intervention approaches are possible and are promising in addressing the problems of women, mothers, neonates and children [28]. A study by UNHCR reported that the Myanmar refugees residing in the Thai-Myanmar border were unable to access adequate health services, especially for mental health and psychological care and HIV/AIDS counselling [24]. Interestingly, the knowledge and practice in relation to ANC were much better among the FDMNs living in the makeshift settlements compared to those living in the host communities. This may be because of the prior existence of health clinics run by an NGO within the settlements. Many of the female FDMNs in the makeshift settlements seemed to have obtained health-related information and care from these clinics. However, the clinics were closed a year prior to our data collection. A study by Matthew and colleagues reported that 82.9% of the Syrian refugee women received ANC and 68.3% of the total care was provided by skilled professionals [29].

In our study, the participants were well informed about the requirement of nutritious food during pregnancy, delivery and after the childbirth. They were also aware about the importance of health care services for the women and children, but they shared that it was difficult for them to afford the health care cost due to their low economic status. The FDMN women knew about the common seasonal health problems including diarrhoea, jaundice, pneumonia, common cold, viral fever, and malaria; however, children still suffer due to the poor living environment, inadequate supply of safe drinking water, and poor health and sanitation in and around the settlement areas. Access to and use of basic health services remains a biggest challenge for people in LMICs, in particular for those living as refugees or asylum seekers [1, 5, 19, 25]. The UNHCR study in Thailand also reported that the Myanmar refugees had inadequate access to essential services and other challenges including overcrowding in the camps, insufficient food rations, lack of non-food items, limited movement outside the camp and risk of exploitation, arrest and deportation [24]. The study by Falb and colleagues reported that overall, 9.6% of women living with a partner along the Thai-Myanmar border suffered conflict victimization and 7.9% of women experienced intimate partner violence (IPV) in the past year [30]. Further, the findings showed that those women who experienced conflict victimization were almost six times more likely to report past‐year IPV than women who had not experienced conflict victimization [30]. These findings suggest the critical needs for developing the integrated approaches to address the health and non-health problems faced by women living in complex situation like refugee camps.

Evidence shows that the refugees in the African continent also faced similar problems as those living in the Asia and Pacific regions including Bangladesh [26, 27]. A study by Kumssa and colleagues reported that the Somali refugees living in Northeastern Kenya suffered from inadequate supply of food, insecurity, poor sanitation, lack of water, congestion, diseases outbreak such as diarrhoea, common cold and malaria, problem of shelter, and restriction in physical movement [26]. Refugees also had problems of discrimination, lack of educational opportunities for children, lack of employment opportunities and negligence from the agencies concerned [26, 27]. Our study found that access to and utilization of basic health services including services for ANC, safe delivery, access to drugs and medication, neonatal and child health care were key challenges for the FDMNs living in Bangladesh. Previous studies conducted among the refugees and migrants in Thailand [31] and countries of Southeast Asia [32] also highlighted similar problems. A study by Koning and colleagues reported that the Myanmar refugee mothers living in Thai-Myanmar border who experienced human right violations had significantly higher odds of unmet ANC need, suffered birth complications, low-birth of new-born babies and poor maternal and child health compared to those who did not experience human right violation [31]. Similarly, the WHO Southeast Asia Regional Office (SEARO) (2018) reported that the refugees and migrants in the region suffer a range of reproductive and maternal health related problems [32].

The findings reported from our current study as well as the ones reported by other studies suggest the importance of developing intervention approaches from the health and human rights perspective, that are context specific, culturally appropriate, and acceptable for those living in refugee camps and temporary settlements. In the light of such needs, the host countries and the development partners including WHO, UNHCR, IOM and ILO have been providing a range of services to the refugees and migrants [32]. For example, the Thai government in recent years is putting their efforts to ensure universal access to health services including reproductive health for those migrants as well as undocumented nationals living in Thailand. The countries in the regions including Sri Lanka, Malaysia, India, Indonesia etc. have developed policies and frameworks that protect rights of migrants and refugees in terms of access to and use of essential health services [32]. However, the policy frameworks and guidelines seem to be considering migrants and refugees in a similar manner. The refugees are distinct to migrant workers who are more vulnerable to several human rights and health related issues, so these populations should get special attention and consideration in order to ensure their right to access and use of essential health services including reproductive and maternal health [33, 34]. Having such consideration can lead to preventing the reproductive health complications, maternal, neonatal and child deaths and improving overall health of women and mothers living in the challenging settings like refugee camps and temporary settlements.

To achieve the 2030 Sustainable Development Goals (SDGs) agenda—leave no one behind [35]—it is critically important that the health needs of refugees and migrants be adequately addressed [33]. The policies and frameworks [36, 37] developed at global, regional and country levels need to be translated into good practice in the efforts of providing services to refugees and migrants living in challenging situation. Bangladesh is not a signatory to the 1951 Refugee Convention or its’ 1967 Protocol and neither is it a party to the 1954 and 1961 Convention on Statelessness [5]. In the absence of a specific refugee policy in Bangladesh and politicization of the refugee situation, integration of Rohingya refugees has always been a challenge [5, 12]. Despite these, there has been a critical need for finding a durable solution to addressing not only the health, but also the non-health problems of people residing in refugee camps and temporary settlements.

To our knowledge, this is the first study ever that has been conducted by using cross-sectional data to provide current scenario, accessibility and utilization of health services among FDMNs in Bangladesh. However, this study has a few limitations. There was a possibility of under or over-reporting on the health and related information since the data were collected by self-report. Information on sensitive topics such as the use of FP methods, sexual and reproductive health issues, and GBV that may result in concealing of accurate information, particularly in the case of interviewing women participants. However, we tried our best to have the true information by assigning female interviewers for female participants.

## Conclusion

The overall health situation of the FDMNs was poor and they had inadequate access to and utilization of health services. The FDMNs did not have adequate knowledge related to HIV/AIDS and TB symptoms and transmission, unsafe sex, use of family planning methods, emergency contraceptives, and gender-based violence. Given the poor health situation as well as poor access to and use of health services among the FDMNs, we recommend continuous essential health and nutrition support programs at a larger scale with the particular focus on improving the easy access to maternal and child health services that promote ANC, PNC, and safe delivery. When these supports are provided, special attention needs to be paid to ensure cultural and religious consideration. Furthermore, male participation also be encouraged in order to improve particularly gender equity and family planning. Additionally, more interventions and supports for women empowerment are recommended in order to allow the female FDMNs to make decisions themselves in relation to their lives.

## Data Availability

All relevant data are presented in this paper. Additional data could be available upon request to the corresponding author.

## Abbreviations

ANC: antenatal care
FDMNs: Forcibly Displaced Myanmar Nationals
FGD: focus group discussions
GBV: gender-based violence
IDPs: internally displaced persons
IOM: International Organization for Migration
PNC: post-natal care
UNHCR: United Nations High Commissioner for Refugees
